# Polypyrrole Nanotubes and Their Carbonized Analogs: Synthesis, Characterization, Gas Sensing Properties

**DOI:** 10.3390/s16111917

**Published:** 2016-11-15

**Authors:** Jitka Kopecká, Miroslav Mrlík, Robert Olejník, Dušan Kopecký, Martin Vrňata, Jan Prokeš, Patrycja Bober, Zuzana Morávková, Miroslava Trchová, Jaroslav Stejskal

**Affiliations:** 1 Department of Physics and Measurements, University of Chemistry and Technology Prague, Prague 6 CZ–166 28, Czech Republic; Jitka.Kopecka@vscht.cz (J.K.); martin.vrnata@vscht.cz (M.V.); 2 Centre of Polymer Systems, University Institute, Tomas Bata University in Zlin, Zlin, CZ–760 01, Czech Republic; mrlik@ft.utb.cz (M.M.); olejnik@ft.utb.cz (R.O.); 3 Faculty of Mathematics and Physics, Charles University in Prague, Prague 8, CZ–180 00, Czech Republic; jprokes@semi.mff.cuni.cz; 4 Institute of Macromolecular Chemistry, Academy of Sciences of the Czech Republic, Prague 6, CZ–162 06, Czech Republic; bober@imc.cas.cz (P.B.); moravkova@imc.cas.cz (Z.M.); trchova@imc.cas.cz (M.T.); stejskal@imc.cas.cz (J.S.)

**Keywords:** polypyrrole nanotube, carbon nanotube, carbonization, functionalized nanotube, heptane detection

## Abstract

Polypyrrole (PPy) in globular form and as nanotubes were prepared by the oxidation of pyrrole with iron(III) chloride in the absence and presence of methyl orange, respectively. They were subsequently converted to nitrogen-containing carbons at 650 °C in an inert atmosphere. The course of carbonization was followed by thermogravimetric analysis and the accompanying changes in molecular structure by Fourier Transform Infrared and Raman spectroscopies. Both the original and carbonized materials have been tested in sensing of polar and non-polar organic vapors. The resistivity of sensing element using globular PPy was too high and only nanotubular PPy could be used. The sensitivity of the PPy nanotubes to ethanol vapors was nearly on the same level as that of their carbonized analogs (i.e., ~18% and 24%, respectively). Surprisingly, there was a high sensitivity of PPy nanotubes to the *n*-heptane vapors (~110%), while that of their carbonized analog remained at ~20%. The recovery process was significantly faster for carbonized PPy nanotubes (in order of seconds) compared with 10 s of seconds for original nanotubes, respectively, due to higher specific surface area after carbonization.

## 1. Introduction

Since carbon nanotubes (CNTs) appeared to be very attractive for volatile organic compounds detection, many research groups focused on the development of the various sensors [1,2,3,4,5,6,7]. It was observed that especially defects and/or impurities, such as heteroatoms, present in nanotubes are responsible for their detection ability, since they modify generally low chemical interaction of CNTs with gas or vapor analytes [2,8]. The neat CNTs are able to detect only molecules with electron-donating (e.g., NH_3_) or electron-accepting (e.g., NO_2_) properties, but, in general, for the detection of such weakly absorbed molecules on the surface their change of the resistance (i.e., sensitivity) is small [9]. On the contrary, the functionalized nanotubes exhibit higher molecular reactivity, therefore the development of new carbonaceous materials with controlled morphology is a promising research direction in sensing applications [10,11,12,13,14]. Polypyrrole (PPy) is also known to be a promising material for gas sensors detecting vapors of organic solvents [15]. Another interesting aspect is represented by the possibility to study properties of PPy-based sensors by impedance spectroscopy [16].

Conducting polymers, such as PPy [17,18,19], have been shown to produce nitrogen-containing carbons when exposed to temperature above 600 °C in an inert atmosphere. It is important to stress that the morphology is retained during this process but the specific surface area is likely to increase. An extensive review concerning the carbonization of these two polymers has recently been published [20].

Polypyrrole is usually prepared by the oxidation of pyrrole with iron(III) salts [21,22] or ammonium peroxydisulfate [23]. Polypyrrole typically has a globular morphology [21]. When prepared in the presence of so-called structure-guiding agents, such as methyl orange, PPy is obtained as nanotubes [19,24,25,26,27,28,29]. It is of interest if the difference in nano-scale morphology, globular or nanotubular, would be reflected in sensing applications. 

In present study, globular and nanotubular PPy has been prepared and subsequently converted to nitrogen-containing CNTs by exposure to elevated temperature in an inert atmosphere. The vapor response of both the original and carbonized PPy was investigated for two organic solvents, polar ethanol and non-polar *n*-heptane, by using evaluation of resistance changes.

## 2. Materials and Methods

### 2.1. Preparation

Globular PPy was prepared by chemical polymerization of pyrrole monomer with iron(III) chloride hexahydrate at equimolar ratio in water. Molar concentrations of both reactants were 422 mM, total volume of reaction mixture was 379 mL. The stirred reaction mixture was kept at 5 °C for 24 h. The precipitated PPy was separated by filtration, rinsed with water and acetone, and dried at 40 °C in vacuo. 

Polypyrrole nanotubes were synthesized in similar manner in the presence of structure-guiding additive, methyl orange, and sodium 4-[4-(dimethylamino)phenylazo]-benzenesulfonate. 200 mL of 2.5 mM solution of methyl orange in distilled water and 700 μL of pyrrole were mixed. Then solution of 10 mmol iron(III) chloride hexahydrate dissolved in 23 mL distilled water was added drop-wise during two hours. Both solutions were cooled to 5 °C before mixing and kept at this temperature afterwards. Molar concentrations of reactants thus were 45 mM pyrrole, 45 mM iron(III) chloride hexahydrate, and 2.2 mM methyl orange. After 24 h, precipitated PPy nanotubes were isolated by filtration, and purified by Soxhlet extraction using acetone until the extract was colorless. Polypyrrole nanotubes were dried as above. Both samples were converted to PPy bases [30] by overnight immersion in excess of 1 M ammonium hydroxide, rinsed with acetone, and dried. 

### 2.2. Carbonization of Polypyrrole

Thermogravimetric analysis was used at first as an analytical tool of PPy carbonization. This was performed in 50 cm^3^·min^−1^ nitrogen flow at a heating rate of 10 °C·min^−1^ with a TGA 7 Thermogravimetric Analyzer (Perkin Elmer, Waltham, MA, USA). A comparative experiment in air has also been done.

In a preparative carbonization, 5 g of PPy nanotubes or globular PPy bases were heated in an inert nitrogen atmosphere to 650 °C in an electric oven. The selection of this particular temperature was made according previous experiments on polyaniline and PPy. In case of polyaniline it follows from the evolution of the infrared and Raman spectra that after carbonization at 650 °C, the G and D bands characteristic of a carbon material are well developed and the residue of the sample is close to 60 wt %. It has been proven also for nanotubular PPy derived carbon nanotubes [20]. When the carbonization temperature was lower, the carbonization was not complete. At higher temperatures, the yield of carbonized product is substantially reduced.

The heating was switched on, and the temperature increased at 10 °C·min^−1^ rate. After the target temperature was reached, the heating was switched off, and the residue was left to cool down in the flowing nitrogen stream.

### 2.3. Characterization

Infrared spectra in the range of 400–4000 cm^−1^ were recorded at 64 scans per spectrum at 2 cm^−1^ resolution using a fully computerized NEXUS 870 FTIR Spectrometer (Thermo Fisher Scientific, Waltham, MA, USA) with DTGS TEC detector (Thermo Fisher Scientific). Samples were dispersed in potassium bromide and compressed into pellets. Raman spectra excited with a diode 785 nm laser were collected on a Renishaw inVia Reflex Raman spectroscope. A research-grade Leica DM LM microscope (Leica Microsystems, Wetzlar, Germany) with an objective magnification 50× was used to focus the laser beam. The scattered light was analyzed by the spectrograph with a holographic grating 1200 lines mm^−1^. A Peltier-effect cooled CCD detector (576 × 384 pixels) registered the dispersed light. To avoid degradation of the samples by the laser beam, a reduced beam power was always used. Transmission electron microscope (TEM) JEOL JEM 2000 FX (JEOL, Tokyo, Japan) and scanning electron microscope (SEM) JEOL 6400 (JEOL, Tokyo, Japan) were used to assess the morphology. Specific surface area was determined by nitrogen adsorption using a Gemini VII 2390 Analyzer (Micrometrics Instruments Inc., Norcross, GA, USA).

Room temperature conductivity of PPy nanotubes was determined on pellets compressed at 700 MPa by a four-point method in the van der Pauw arrangement using a Keithley 220 Programmable Current Source, a Keithley 2010 Multimeter (Keithley Instruments, Solon, OH, USA) as a voltmeter and a Keithley 705 Scanner (Keithley Instruments) equipped with a Keithley 7052 Matrix Card (Keithley Instruments). The conductivity of globular PPy and carbonized PPy was estimated on powders placed between two conducting pistons by two-probe method with applied pressure of ca. 23 kPa and using a Keithley 6517 electrometer (Keithley Instruments). 

### 2.4. Vapor Response

Polypyrrole nanotubes, both original and carbonized form, were dispersed in 50 mL of deionized water containing 0.1 M of sodium dodecyl sulfate (SDS) surfactant (Sigma Aldrich, St. Louis, MO, USA) and 0.14 M of 1-pentanol (Sigma Aldrich), respectively. The concentration of nanotubes in the suspension was 0.3 wt %. The suspension was homogenized in an ultrasonic apparatus (UZ Sonopuls HD 2070, Bandelin, Germany) for 5 min at ca. 50 °C. Polypyrrole nanotube networks were prepared by vacuum filtration of suspension thought nonwoven polyurethane membrane, composed of polyurethane straight fibers with average diameter 0.14 ± 0.09 µm. The fibers’ surface was smooth and the main pore size was around 0.2 µm. Thus, prepared network on polyurethane support was rinsed several times with deionized water and methanol. After drying, this composite structure was tested as a layer sensitive to volatile organic compounds when exposed to the vapors of *n*-heptane and ethanol (having nearly the same vapor pressure at room temperature but with different polarity). For sensitivity testing, laboratory air was used as the reference gas. Aqueous suspensions of globular PPy in 0.4 wt % concentration were similarly prepared and processed. 

The stripes 5 × 20 mm^2^ made of active components deposited on polyurethane supports were placed on a planar holder with copper electrodes fixed on both sides of the stripe by a screw mechanism. Time-dependent electrical resistance was measured along the specimen length by the two-point technique using a multimeter Keithley 6517B (Keithley Instruments) during adsorption (analyte-on phase) and desorption (analyte-off phase). The holder with the specimen was transferred into an air-tight conical flask containing saturated vapors of the respective solvent at atmospheric pressure and 25 °C. Under these conditions, the saturated vapor of ethanol has a concentration of 7.7 vol % and the corresponding value for *n*-heptane is 6.0 vol %. After 6 min of measurement the holder was removed from the flask and, for the next 6 min, the sample resistance was measured in laboratory air in the desorption mode until steady state. The sensitivity of sensors, a relative change in resistivity is defined as *S* [%] = (*R*_g_ − *R*_a_)/*R*_a_ × 100, where *R*_a_ is the resistance in air under laboratory conditions and *R*_g_ is the resistance of the specimen exposed to organic vapor. 

## 3. Results and Discussion

**Figure 1:**
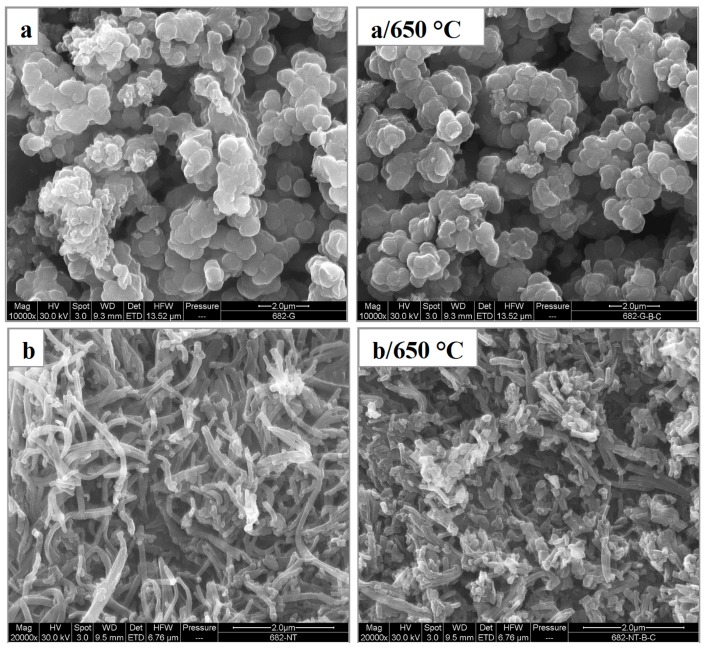
Scanning electron micrographs of (**a**) original globular polypyrrole and (**b**) polypyrrole nanotubes. They are depicted before- (left column) and after- carbonization at 650 °C (right column).

**Figure 2:**
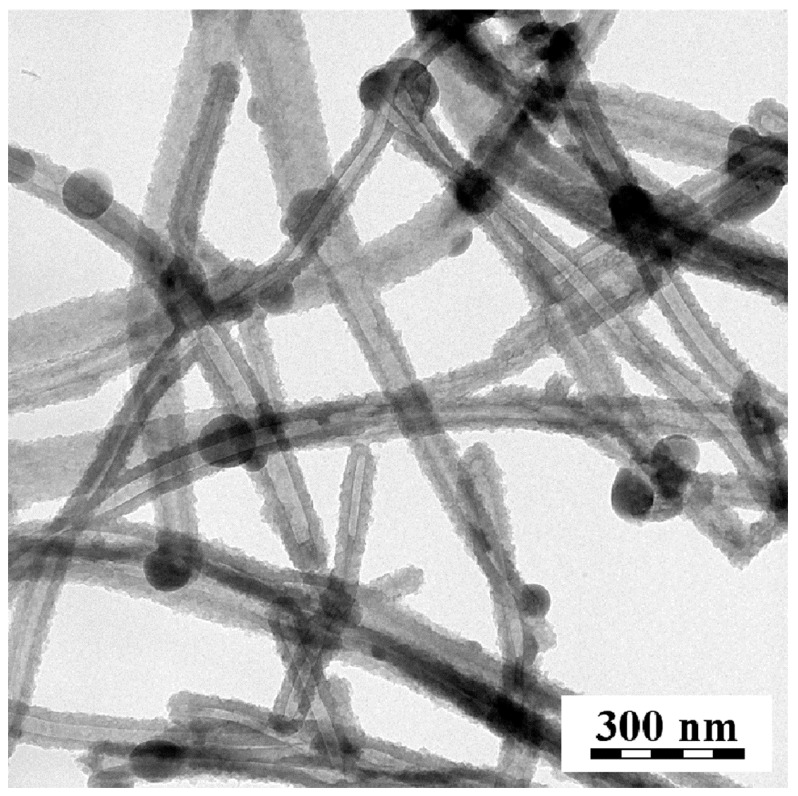
Illustration of nanotubular morphology by transmission electron microscopy.

The classical preparation of PPy yields a product with globular morphology [21] (Figure 1a left). Its carbonization proved that the morphology is retained when this process is carried out in an inert atmosphere (Figure 1a right). The introduction of methyl orange to the reaction mixture results in a dramatic change in polymer morphology, and PPy nanotubes are obtained instead (Figure 1b left). The cavity inside the nanotubes is demonstrated by using transmission electron microscopy (Figure 2).

**Figure 3:**
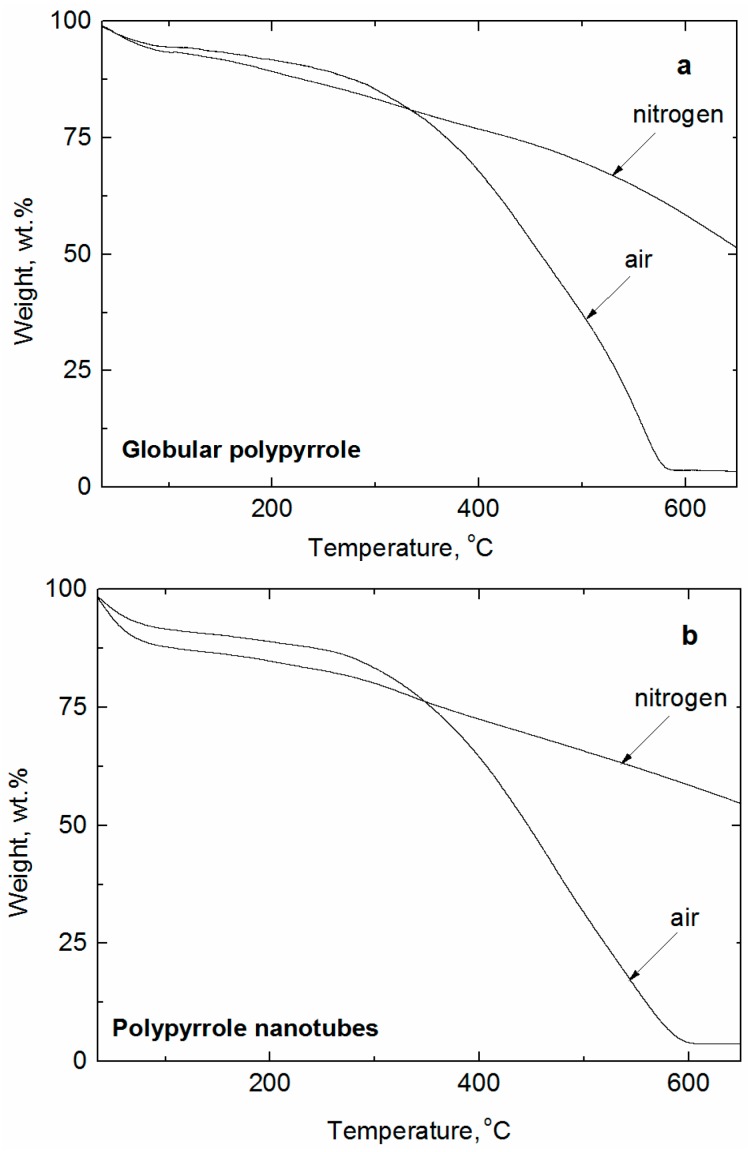
Thermogravimetric analysis of (**a**) globular polypyrrole and (**b**) polypyrrole nanotubes in air and in nitrogen.

The nanotubular structure is damaged but not destroyed after carbonization (Figure 1b right). The shrinkage is the consequence of the loss of mass during the exposure to elevated temperature (Figure 3).

The conductivity of PPy obtained by oxidative polymerization of pyrrole with iron(III) chloride is usually around units S·cm*^−^*^1^ [31,32]. In the presented case, the conductivity of globular PPy was of the order of 10*^−^*^2^ S·cm*^−^*^1^ (Table 1). By changing the morphology from globules to nanotubes, the conductivity increased to 60 S·cm*^−^*^1^. After the deprotonation with ammonium hydroxide the conductivity decreases by several orders of magnitude due to conversion of conducting PPy to less conducting PPy base. Polypyrrole bases have originally been intended for the application in electrorheology [33,34,35] but, for that purpose, the conductivity of the nanotubular form was too high. For that reason, the samples have been tested in the present study for sensing properties, where the level of conductivity is suitable. 

### 3.1. Analytical Carbonization

To get a deeper insight into the process of carbonization, thermogravimetric analysis was performed both in air and nitrogen atmosphere. The analysis in air illustrates the complete destruction of PPy between 550 °C and 600 °C (Figure 3a). There is no significant difference in the thermal stability between globular and nanotubular forms of PPy. A residue of ≈5 wt % is most likely represented by iron oxides produced from the oxidant, iron(III) chloride.

In inert nitrogen atmosphere, however, the residue is in both cases above 50 wt % at 650 °C. Also here, there is no substantial difference in the stability of globular and nanotubular PPy (Figure 3b). This is logical, because the thermal stability is established by molecular, rather than supramolecular, structure. 

### 3.2. FTIR Spectroscopy

#### 3.2.1. Polypyrrole Bases

**Figure 4:**
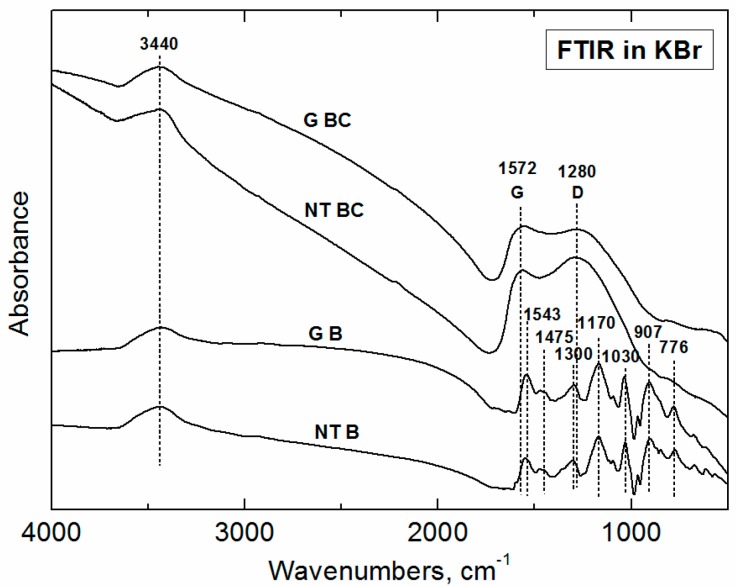
FTIR spectra of original globular (G) and nanotubular (NT) polypyrrole bases before (B) and after (BC) carbonization.

Infrared spectra of granular and nanotubular PPy bases (Figure 4) are close to each other and correspond well to the spectra of PPy bases described in the literature [23]. We observe a broad absorption band at wavenumbers above 2000 cm^−1^, and the band at about 1700 cm^−1^ which corresponds to the presence of a carbonyl group formed by the nucleophilic attack of pyrrole by water during the preparation [21,23]. The band at 1572 cm^−1^ is assigned to C–C stretching vibrations in the pyrrole ring, the band at 1475 cm^−1^ to C–N stretching vibration in the ring. A broad band attributed to C–H or C–N in-plane deformation modes with a maximum at 1300 cm^−1^ is well detected in the spectra. In the region of the C–H and N–H in-plane deformation vibrations from 1250 to 1000 cm^−1^, we observe a maximum at 1170 cm^−1^ in the spectra of PPy base. The bands corresponding to the C–H and N–H in-plane deformation vibrations are situated at 1030 cm^−1^ and to C–C out-of-plane ring-deformation vibrations at 965 cm^−1^. The C–H out-of-plane deformation vibrations of the pyrrole ring (at about 907 cm^−1^) and of the C–H out-of-plane ring deformations (at about 776 cm^−1^) are present in the spectra of the PPy base.

#### 3.2.2. Carbonized Materials

In the FTIR spectra of carbonized PPy bases, we observe a local maximum at 1572 cm^−1^, and a broad band with a maximum at about 1280 cm^−1^. The first band emerged from the C–C stretching vibrations in the pyrrole ring, the second from the C–N in-plane deformation modes. The shape of the spectra is close to that of the spectra of a carbon-like material with the Raman-active D and G bands, which are usually inactive in FTIR spectra. In disordered samples, however, they become IR-active because of symmetry-breaking of the carbon network, but they are rather weak and the spectra are flat and almost featureless.

### 3.3. Raman Spectroscopy

#### 3.3.1. Polypyrrole Bases

**Figure 5:**
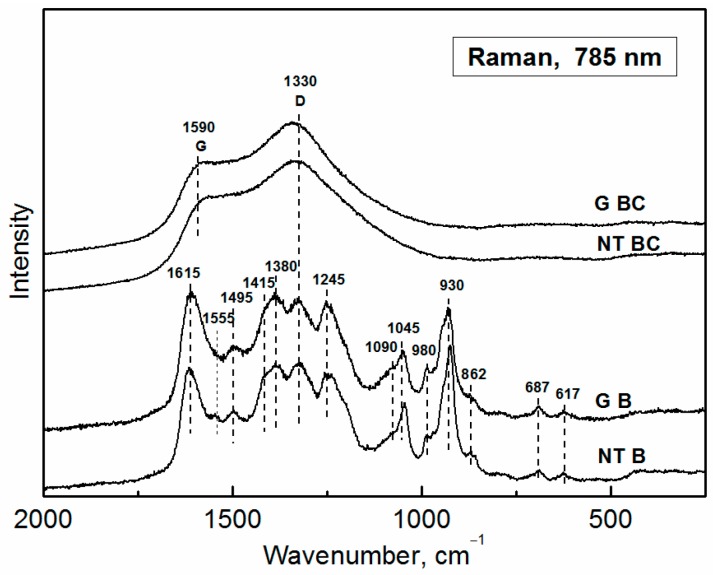
Raman spectra of original globular (G) polypyrrole bases and polypyrrole nanotubes (NT) before (B) and after carbonization (BC).

Raman spectroscopy is well suited to characterize the progress of carbonization (Figure 5). Raman spectra of powdered samples have been recorded with excitation wavelength 785 nm. The spectra of globular and nanotubular PPy bases differ in elevated intensity and narrower shape of the band located at 930 cm^−1^ (C–H out-of-plane deformation vibrations of dication-bearing unit [36]), the presence of band at 1555 cm^−1^, and a shoulder around 1415 cm^−1^ in the case of nanotubular PPy. The PPy base bands are located at 1615 cm^−1^ (C=C stretching in the pyrrole ring), 1495 cm^−1^ (C=N stretching vibrations in the pyrrole ring), 1390 cm^−1^ (C–H and N–H bending, 1330 cm^−1^ (C–C stretching of neutral units), 1245 cm^−1^ (antisymmetric C–H bending), 1045 cm^−1^ (in-plane ring-deformation vibrations) with a shoulder at 1090 cm^−1^ (C–H, N–H and out-of-plane ring-deformation vibrations), 980 cm^−1^ (C–C deformation vibrations in neutral rings), 687 cm^−1^, and 617 cm^−1^ (ring-deformation vibrations).

#### 3.3.2. Carbonized Materials

In the Raman spectra of carbonized PPy the band at 1590 cm^−1^ (emerged from the C=C stretching vibrations of the pyrrole ring) and the broad band centered at 1330 cm^−1^ (emerged from the C–C stretching vibrations of the pyrrole ring) can be observed. These bands can be considered as G-band (“graphitic” band, C=C stretching vibrations of any pair of sp^2^ sites) and D-band (“disorder” band, breathing of aromatic rings activated by any defect including a heteroatom), bands defined for graphitic material [37] and proved to be usable for nitrogen-doped graphitic material. The spectrum corresponds to a disordered nitrogen-containing graphitic material.

### 3.4. Vapor Response

**Figure 6:**
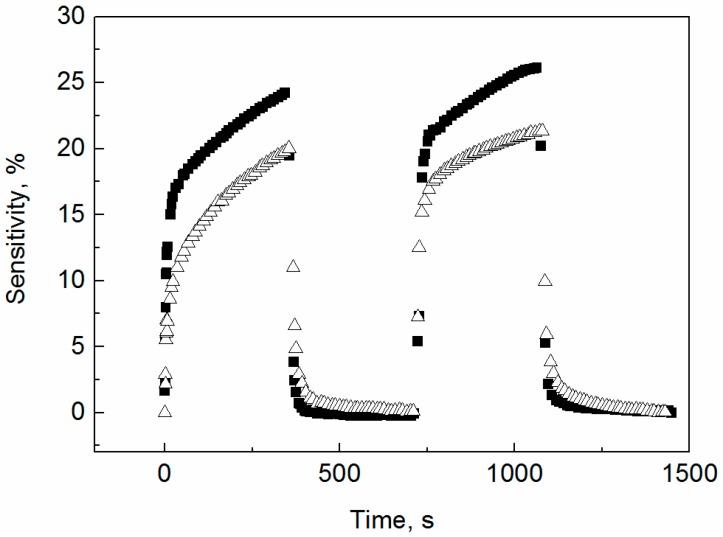
The response profile during two adsorption/desorption cycles in the presence of ethanol (7.7 vol %) for (△) polypyrrole nanotubes and (■) carbonized polypyrrole nanotubes.

**Figure 7:**
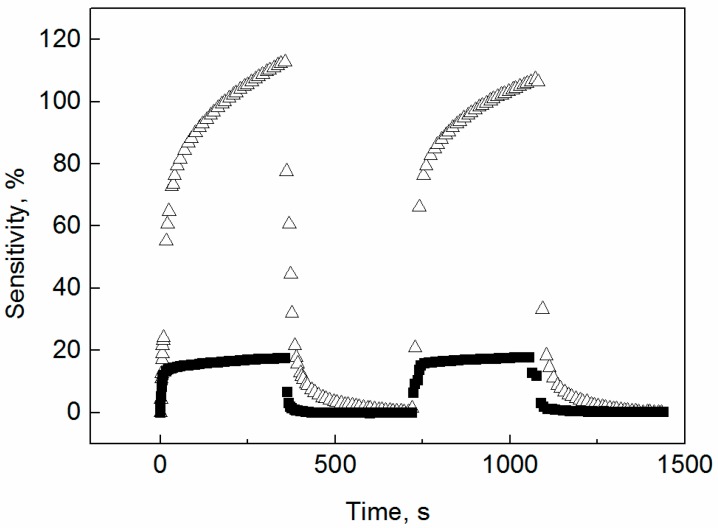
The response profile during two adsorption/desorption cycles in the presence of *n-*heptane (6.0 vol %) for (△) polypyrrole nanotubes and (■) carbonized polypyrrole nanotubes.

In the case of globular PPy samples, the resistivity of the prepared materials was too high, thus the vapor response properties of corresponding sensors were not measurable. The response properties of both PPy nanotubes as well as their carbonized forms were obtained (Figure 6 and Figure 7). When detecting ethanol vapors (Figure 6), the sensitivity (i.e., a relative increase in resistance) of both nanotubular samples differs only slightly (i.e., 18% for non-carbonized PPy and 24% for a carbonized analog). When detecting *n*-heptane (Figure 7), the significantly better sensitivity of 110% was determined for the original PPy nanotubular base, while the carbonized ones exhibit only a 20% response. The high sensitivity to *n*-heptane is remarkable because typical values reported in the literature for detection of alkanes are of the order of units of a percent [38]. We can speculate that the high sensitivity of as-synthesized of PPy to *n*-heptane is connected with the reduction of humidity level in the sample and consequent increase in its resistivity. Another interpretation of this phenomenon can be made according to basic theory of sensing mechanisms on conducting polymers [39,40,41,42]: Doping and undoping play key roles in the sensing mechanism of conducting polymer based sensors. Their doping level can be altered by transferring electrons from or to the sensitive layer. All π- or σ-electron donating gases can be detected. Our analyte (*n*-heptane) probably acts as an σ-electron donor.

There is still one important trend observable (Figure 6 and Figure 7), a significantly faster response of carbonized nanotubes on the change of the vapor environment especially in recovery process for both investigated solvents. This is probably due to structural changes during carbonization which are connected with increasing material porosity.

To determine the detection limit for ethanol and *n*-heptane, we can take into account a conventional definition that the minimum detectable signal (i.e., detection limit) of a sensor should be calculated as a value of input quantity which causes change of output quantity three times higher than it is the effective noise of output quantity. When evaluating the meta-data for Figure 6 and Figure 7, we observed that the level of noise of sensor output is lower for carbonized polypyrrole nanotubes than for as-prepared polypyrrole nanotubes (this is a positive aspect of the former sensors). With respect to meta-data and the above-mentioned definition the detection limits were calculated as follows: 3000 ppm of ethanol on carbonized PPy nanotubes; 5000 ppm of ethanol on PPy nanotubes; and 10,000 ppm of *n*-heptane on carbonized PPy nanotubes; 5000 ppm of *n*-heptane on PPy nanotubes. 

A comparison of our sensitivity results with those found in the literature is included in Table 2. It is limited to detection of two relevant classes of compounds—alkanes and alcohols—on PPy nanotubes or their carbonized analogs. We have converted various expressions of sensitivity reported in original references to a quantity defined as the relative change in the resistivity, S = (∆R/R_0_) × 100 [%].

For alkanes detected on PPy (Table 2), there is reported detection of 1040 ppm of propane/butane with sensitivity 55% [39], and an unknown concentration of hexane with sensitivity 0.8% [38]. In the present case, it was 60,000 ppm of *n*-heptane with sensitivity 110%. As for alkanes detected on carbon nanotubes, only response to the saturated vapor is reported (i.e., 905,000 ppm at 25 °C) of iso-pentane which yielded sensitivity of 20.3%, 12.6%, 20.6%, and 12% at various conditions [3]. The present result is 60,000 ppm of *n*-heptane with sensitivity of 20%.

As for alcohol vapors detected on PPy, there are mainly responses reported to vapors saturated at 25 °C (their concentration is in the order of 10,000–100,000 ppm) in dependence on the number of carbon atoms in the alcohol molecule. The sensitivity varies from 0.22% to 37.5% [15,38,40,41,42]. The present result is 18% for 77,000 ppm of ethanol. As for alcohol vapors detected on carbon nanotubes—for saturated methanol vapor there was reported sensitivity from 12.9% to 46.6% [3,5]—one paper presents significantly higher sensitivity, i.e., 429% and 4500% for saturated methanol vapor [4], while the result of this study is 24% for 77,000 ppm of ethanol.

To conclude, the preliminary results related to detection of *n*-heptane are significantly better than the average of those reported elsewhere. When comparing the response to alcohol vapors, there is a wide range of sensitivities; and they should be assessed case-by-case. The values obtained in this study are approximately in the middle of interval of reported sensitivities [3,5]. The detection of both alkanes and alcohol vapors will be the subject of our further systematic research.

## 4. Conclusions

During the oxidation of pyrrole with iron(III) chloride, globular morphology of PPy is transformed to nanotubes by addition of methyl orange. PPy bases obtained after deprotonation convert to nitrogen-containing carbon by heating to 650 °C in inert atmosphere. The original morphology is preserved after carbonization. Conductivity of PPy was reduced after the conversion to bases as well as after subsequent carbonization. The conductivity of globular form becomes too low for the application in sensors. The nanotubular PPy base, however, was demonstrated to respond to ethanol or *n*-heptane vapors by the change in resistivity. The sensitivity of the original nanotubular base to *n*-heptane reached 110%, which is a unique result, and even the carbonized analog maintained a 20% sensitivity. The recovery was faster in the carbonized PPy. Organic vapor may reduce the humidity in the samples and thus cause a consequent increase in the resistivity.

## Figures and Tables

**Table 1:** Conductivity and specific surface area of globular and nanotubular PPy salts, bases, and their carbonized analogs.

Sample	Conductivity (S·cm^−1^)			Specific Surface Area (m^2^·g^−1^)		
	Salt	Base	Carbonized Base	Salt	Base	Carbonized Base
PPy nanotubes	60 ^a^	6.7 × 10^−2^ ^a^	6.7 × 10^−6^	75	63	211
Globular PPy	0.011	3.4 × 10^−5^	1.4 × 10^−7^	26	25	150

^a^ Measured on pellets compressed at 530 MPa pressure. Pellets could not be prepared from other samples. Their conductivity was estimated in a powdered state. Such conductivities are usually one to two orders of magnitude lower compared with those of the pellets.

**Table 2:** Overview of sensitivity of sensors based on PPy nanotubes, nitrogen-containing carbons, and MWCNT. The detected analytes are alkanes and alcohol vapors.

Sensor Design	Detection Conditions				
Sensitive Material	Analyte	Concentration	*S* (%)	Temp./Hum. (°C)/(% RH)	References
Sensors of this work					
PPy nanotubes deprotonated	ethanol	saturated vapors at 25 °C	18	25/0	This work
	*n*-heptane		110		
PPy nanotubes carbonized	ethanol		24		
	*n*-heptane		20		
Polypyrrole based sensors ^a^					
PPy/sulfate	propane/butane	1040 ppm	55	27/35	[39]
PPy/Cl^–^	hexane	-	0.8	100/0	[38]
	methanol		5		
PPy/ClO_4_^−^	*iso*-butanol	saturated vapors at 25 °C	15.5	25/0	[15]
	ethanol		10.4		
	*iso*-propanol		15.8		
	*n*-pentanol		11.2		
PPy/PF_6_^−^	*iso*-butanol		0.5		
	ethanol		3.2		
	*iso*-propanol		0.6		
	*n*-pentanol		1.1		
PPy/CF_3_SO_3_^−^	*iso*-butanol		3.0		
	ethanol		7.5		
	*iso*-propanol		4.4		
	*n*-pentanol		1.3		
PPy/camphorsulfonate	*iso*-butanol		6.1		
	ethanol		5.8		
	*iso*-propanol		5.7		
	*n*-pentanol		5.1		
PPy/*p*-toluenesulfonate	methanol	-	18	-	[40]
	ethanol		10		
PPy/3-nitrobenzenesulfonate	methanol		11		
	ethanol		6		
Nanostructured PPy/ClO_4_^−^	methanol	11% wt. of VOC in *n*-hexane	2.9	120/0	[41]
	ethanol		0.58		
	*n*-propanol		0.22		
	*iso*-propanol		0.18		
Nanostructured PPy/*p*-toluenesulfonate	methanol		1.5		
	ethanol		0.75		
	*n*-propanol		*–*		
	*iso*-propanol		*–*		
PPy/PCP ^a^	methanol	*–*	7	*–*/0	[42]
	ethanol		7		
PPy/PEO	methanol		3.5		
	ethanol		5.5		
PPy/PMMA	methanol		65		
	ethanol		20		
PPy/PVAL	methanol		14		
	ethanol		14		
PPy/PVAc	methanol		27.5		
	ethanol		37.5		
CNT based sensors					
MWCNT	*iso*-pentane	saturated vapors at 25 °C	20.3	25/60	[3]
	methanol		13.6		
MWCNT/PMMA	*iso*-pentane		12.6		
	methanol		14.7		
MWCNT/PMMA	methanol	saturated vapors at 25 °C	429	*–*/0	[4]
	hexane		*–*		
Surface modified MWCNT/PMMA	methanol		4500		
	hexane		*–*		
MWCNT	*iso*-pentane	saturated vapors at 25 °C	20.6	25/60	[5]
	methanol		12.9		
Oxidized MWCNT	*iso*-pentane		12.0		
	methanol		46.6		

^a^ Definition of abbreviations: PCP—polycaprolactone, PEO—poly(ethylene oxide), PMMA—poly(methyl methacrylate), PVAL—poly(vinyl alcohol), PVAc—poly(vinyl acetate), MWCNT—multi-wall carbon nanotubes, CVD—chemical vapor deposition, VOC—volatile organic compounds.
